# Competition between apex predators? Brown bears decrease wolf kill rate on two continents

**DOI:** 10.1098/rspb.2016.2368

**Published:** 2017-02-08

**Authors:** Aimee Tallian, Andrés Ordiz, Matthew C. Metz, Cyril Milleret, Camilla Wikenros, Douglas W. Smith, Daniel R. Stahler, Jonas Kindberg, Daniel R. MacNulty, Petter Wabakken, Jon E. Swenson, Håkan Sand

**Affiliations:** 1 Department of Wildland Resources and Ecology Center, Utah State University, 5230 Old Main Hill, Logan, UT 84322, USA; 2 Grimsö Wildlife Research Station, Department of Ecology, Swedish University of Agricultural Sciences, 730 91 Riddarhyttan, Sweden; 3 Faculty of Environmental Sciences and Natural Resource Management, Norwegian University of Life Sciences, Postbox 5003, 1432 Ås, Norway; 4 Wildlife Biology Program, Department of Ecosystem and Conservation Sciences, University of Montana, Missoula, MT 59812, USA; 5 Yellowstone Center for Resources, Yellowstone National Park, Box 168, Mammoth Hot Springs, WY 82190, USA; 6 Faculty of Applied Ecology and Agricultural Sciences, Inland Norway University of Applied Sciences, Evenstad, 2480 Koppang, Norway; 7 Department of Wildlife, Fish, and Environmental Studies, Swedish University of Agricultural Sciences, 901 83 Umeå, Sweden; 8 Norwegian Institute for Nature Research, 7485 Trondheim, Norway

**Keywords:** *Canis lupus*, competition, predation, Scandinavia, *Ursus arctos*, Yellowstone

## Abstract

Trophic interactions are a fundamental topic in ecology, but we know little about how competition between apex predators affects predation, the mechanism driving top-down forcing in ecosystems. We used long-term datasets from Scandinavia (Europe) and Yellowstone National Park (North America) to evaluate how grey wolf (*Canis lupus*) kill rate was affected by a sympatric apex predator, the brown bear (*Ursus arctos*). We used kill interval (i.e. the number of days between consecutive ungulate kills) as a proxy of kill rate. Although brown bears can monopolize wolf kills, we found no support in either study system for the common assumption that they cause wolves to kill more often. On the contrary, our results showed the opposite effect. In Scandinavia, wolf packs sympatric with brown bears killed less often than allopatric packs during both spring (after bear den emergence) and summer. Similarly, the presence of bears at wolf-killed ungulates was associated with wolves killing less often during summer in Yellowstone. The consistency in results between the two systems suggests that brown bear presence actually reduces wolf kill rate. Our results suggest that the influence of predation on lower trophic levels may depend on the composition of predator communities.

## 1. Introduction

Understanding the influence of top-down and bottom-up effects on ecosystem regulation is a central focus of ecology (e.g. [1–3]). Although the strength of top-down and bottom-up effects on prey abundance often varies through time [4,5], predation is an important driver of prey population dynamics [6,7]. The composition of predator communities can have profound effects on prey abundance [5,8,9] and the strength of top-down effects can be altered by competition between sympatric predators at the top level of trophic systems [10].

Interspecific interactions between predators are widespread in nature and play an important role in community structure and stability [11]. Ultimately, such interactions can either weaken or strengthen top-down effects by altering predator densities or predation patterns. Kleptoparasitism by competitors, for example, can negatively impact predator foraging efficiency (e.g. [12]), limiting predator abundance and the impact of predation on prey populations [10]. Alternatively, theft of kills can result in increased predation [13,14], potentially increasing the predator's impact on the prey population. Quantifying how competition between apex predators affects predation dynamics is an important step towards understanding the cascading ecological effects of such interactions.

Kill rate (i.e. the number of prey killed per predator per unit time) is an essential component of predation, yet we still have a limited understanding of how it is influenced by interspecific interactions between apex predators. Here, we analysed how the kill rate of one apex predator and obligate carnivore, the grey wolf (*Canis lupus*), was affected by a sympatric apex predator and omnivore, the brown bear (*Ursus arctos*). Brown bears are efficient, typically dominant scavengers of wolf-killed prey, which has motivated the assumption that wolf kill rate is higher where wolves are sympatric with brown bears [15,16] because they are forced to hunt more often to compensate for the loss of food. Understanding how wolf kill rate is affected by bears is especially important, because these two species are largely sympatric in temperate climates [17], where wolves are usually a dominant predation force that can limit the abundance of prey populations [6].

We used data from two long-term studies in southcentral Scandinavia (SCA), Europe, and Yellowstone National Park (YNP), USA, in a first transcontinental attempt to evaluate the assumption that brown bears cause wolves to kill more often. In both systems, wolf predation has been a central research topic for over 15 years [18,19]. We used kill interval (i.e. the number of days between consecutive ungulate kills) as a measure of kill rate and divided our analyses by season, as wolf kill rates vary throughout the year [18,19]. We predicted that (i) kill interval of SCA wolf packs sympatric with brown bears would decrease across the spring bear den emergence period (March–May) as bears progressively emerged from winter dens; wolf packs allopatric with brown bears should exhibit no such decline. We also predicted that, during summer, (ii) wolf kill interval would be lower for wolf packs that were sympatric, compared to allopatric, with bears in SCA, and (iii) the presence of bears at wolf-killed ungulates would decrease wolf kill interval in YNP, where the species are sympatric.

## 2. Material and methods

### (a) Study areas

#### (i) Scandinavia

Sweden and Norway constitute the Scandinavian Peninsula, referred to as Scandinavia. This part of the study was conducted in southcentral Scandinavia (approx. 100 000 km^2^, elevation 50–1000 m), which primarily consists of intensively managed boreal forest (see [20]). Breeding wolf and brown bear populations coexist only in the northern portion of the study area (61° N, 15° E); wolf packs in the southern and western parts of the study area were outside of the brown bear distribution (60° N, 13° E). The wolf population was estimated at 460 (95% CI = 364–598) in the winter of 2014/2015, with their range restricted to SCA [21]. Here, moose (*Alces alces*) are the main prey for wolves, with roe deer (*Capreolus capreolus*) being secondary prey [18,22]. Moose densities in Scandinavia are among the highest in the world (*x̄* = 2 moose per km^2^) [23].

The Scandinavian brown bear population was estimated at 3300 individuals in 2008 [24] and reaches a density of 3 bears per 100 km^2^ in areas where they are sympatric with wolves [25]. During early summer, ungulate neonate calves are the primary food for Scandinavian brown bears [26], with most moose predation occurring in late May–June [27]. Bears in Scandinavia rarely prey on adult ungulates [28]. Although wolves decrease the temporal variation in ungulate biomass available to scavengers in Scandinavia [29], the extent to which wolf-killed prey contributes to brown bear diet remains unknown.

#### (ii) Yellowstone National Park

Yellowstone National Park (8991 km^2^) is a protected area in northwestern Wyoming, USA, that supports wolf and brown bear populations. The study area was limited to northern Yellowstone, known as the Northern Range (NR; 995 km^2^, elevation 1500–2000 m). Since 2008, the NR wolf population ranged between 34 and 57, with the current minimum number estimated at 42 wolves (Yellowstone Wolf Project 2016, unpublished data). Elk (*Cervus elaphus*) are the main prey for wolves in Yellowstone [19]. Secondary prey species include bison (*Bison bison*), deer (*Odocoileus* spp.), bighorn sheep (*Ovis canadensis*), moose and pronghorn (*Antilocapra americana*).

The brown bear population in the Greater Yellowstone Ecosystem (approx. 37 000 km^2^), which encompasses YNP, was approximately 750 bears in 2014 [30], with NR brown bear density ranging between 5 and 15 bears per 100 km^2^ [31]. Brown bears in YNP scavenge ungulate carcasses, particularly after den emergence in early spring [32]. Wolf-killed ungulates, however, provide scavenging opportunities for brown bears throughout the year [33] and contribute to the relatively high proportion of meat in their diet [34,35]. YNP brown bears frequently usurp carcasses from wolves [36]. They also prey on neonate elk from late May–July [34,37], but rarely kill adult ungulates [38]. American black bears (*Ursus americanus*) are also present in YNP, but there is no record of them usurping wolf-killed ungulates.

### (b) Data collection

#### (i) Scandinavia

Predation studies in SCA occurred during two distinct time periods, hereafter referred to as ‘spring’ and ‘summer’. These studies were conducted from 2001 to 2015 on wolf packs whose territories were sympatric (*N*_spring_ = 8; *N*_summer_ = 4) and allopatric (*N*_spring_ = 9; *N*_summer_ = 8) with brown bears (electronic supplementary material, table S1). Wolves were aerially captured and immobilized according to accepted veterinary and ethical procedures [39,40]. At least one breeding adult in each pack was fitted with a GPS collar (Vectronic Aerospace, Germany) and followed during each study period. Kill interval was measured at the ‘pack’ level in SCA, where wolf packs were often small and the breeding pair was generally the main food provider. Field crews searched for carcasses within a 100 m radius of all ‘clustered’ GPS points, and recorded cause of death, species, age and sex of carcasses found (see [41]; electronic supplementary material, appendix S1). Time of first wolf position within the cluster was used as a proxy for the time of death of wolf-killed prey.

The number and distribution of confirmed brown bear deaths is an established index of brown bear distribution and density in Scandinavia [42,43]. We used data on brown bear deaths, including hunter harvest estimates, to create an index of bear density across Scandinavia (see [44]). Harvest estimates are reliable because bear hunters in Scandinavia are not limited to specific hunting districts, and are required by law to report the kill sites of harvested bears. The index ranged from 0 (i.e. areas with no or sporadic bear presence) to 1 (i.e. areas with the highest bear density). Wolf territories were either located in areas with high (index > 0.8) or very low (index < 0.1) bear density. This natural division allowed wolf territories to be categorized as either ‘sympatric’ or ‘allopatric’ with brown bears.

Prey type was categorized as adult or calf moose in spring, and neonate or non-neonate (i.e. newborn calf or adult/yearling) moose in summer. For both systems, multiple carcasses in a kill event were reduced to a single kill and assigned to the largest prey type. Spring and summer pack size estimates were based on snow tracking of GPS-collared wolves during winter. We calculated moose densities using hunter harvest statistics (number of moose harvested per square kilometre) generated at the municipality level in Norway and the hunting management unit in Sweden. Moose density was calculated as the weighted average density of all management units within a wolf territory, using a 1-year time lag, which has been shown to be a good predictor of moose density [45]. Snow depth measurements (metres) for each spring kill date were obtained from the Swedish Meteorological and Hydrological Institute, using the meteorological station closest to each territory. Most stations were located either inside or within 5 km of the territory boundary, except for two territories where the closest station was within 35 km.

#### (ii) Yellowstone National Park

Studies in YNP took place during summer (1 May–31 July) from 2008 to 2015 on 19 wolves in 10 packs (*N* = 23) (electronic supplementary material, table S1). Monitored wolves (breeding and nonbreeding individuals) were captured and fitted with a GPS collar (Lotek; Newmarket, ON, Canada) following animal handling guidelines of the American Society of Mammalogists [46]. Field crews searched for carcasses within a 400 m^2^ area of all clustered GPS points and recorded cause of death, species, age and sex of carcasses found (see [47]; electronic supplementary material, appendix S1). Time of first wolf position within 100 m of the carcass site was used as a proxy for the time of death of wolf-killed prey. Kill interval was treated independently for each monitored wolf within each pack, and was thus measured at the ‘wolf’ level. We did so because more than one wolf per pack was followed during the YNP studies, and here, pack mates often fed at different kill sites during summer [47]. A wolf was associated with an ungulate kill if it (or its pack) killed the animal, and it was located at least twice within 100 m of the carcass 1 or 3 days after death, for a small or large ungulate, respectively [47].

We classified brown bears as ‘present’ at wolf kills if field crews observed a brown bear, or detected bear sign, at the carcass site. In YNP, bear sign was not diagnostic to species at 85% (*N* = 127/149) of carcasses. For the purposes of this study, we assumed that unknown bear sign was indicative of brown bears because this species was most often sighted at wolf kills (86% (*N* = 139/162) of bear sightings at wolf kills, 1995–2015), and most often observed interacting with wolves at carcasses (89% (*N* = 225/254) of wolf–bear interactions, 1996–2016). Therefore, there was a low risk of attributing black bear presence to brown bear presence. Furthermore, black bears are less likely than brown bears to usurp wolf kills [15,35], and therefore less likely to affect wolf kill interval. Thus, attributing black bear presence to brown bears is likely to underestimate any effect that brown bears might have on wolf kill interval. Prey type was categorized as either large (i.e. elk, bison or moose more than 11 months), small ungulate (i.e. any neonate, or adult deer, bighorn sheep or pronghorn) or unknown. We assumed wolves were scavenging when they visited a carcass that had not been killed by their pack. A ‘scavenging event’ was therefore a carcass scavenged by a wolf between consecutive kills. Pack size was recorded as the maximum number of individuals observed during March, unless pack size declined during the study period; newborn pups were not included in summer pack size estimates for either system. Distance of the kill site to the nearest paved or gravel road, a proxy for human disturbance, was measured in kilometres for both SCA and YNP in ArcGIS v. 10.2.

### (c) Data analysis

We estimated wolf kill interval as the number of days between consecutive ungulate kills per pack in SCA and per wolf in YNP. We calculated kill interval in SCA using moose kills only (moose account for more than 95% of the biomass in their diet [18,22]), and in YNP using kills of all ungulate species [19]. In YNP, we included four kills of unknown ungulate species when calculating the time between consecutive kills (*N* = 544). Once the kill interval was established, we subsequently excluded them from the statistical analyses.

#### (i) Spring wolf kill interval in Scandinavia

To determine how brown bear presence influenced wolf kill interval, we compared how kill interval varied across the spring den emergence period (March–May) between wolf packs that were sympatric and allopatric with bears. We assumed that the effective number of bears increased as the emergence period advanced from March to May, and tested for an interaction between kill date and bear presence. We used observations collected between 1 March and 15 May (*N* = 17), the period when bears emerge from their den. In SCA, the mean date of den emergence was 4 April (6 March–25 April) for males [48] and 20 April (6 March–14 June) for females [49]. We removed one pack year from the dataset; the Kukumäki pack was affected by sarcoptic mange in 2013 and had a kill interval that was substantially longer than average during that study period. Model variables in the candidate model set included bear presence, Julian kill date (61–133), pack size (2–9), prey type, moose density (0.006–0.39), distance from the kill site to the nearest road (0.004–1.15 km) and snow depth (0–0.96 m).

#### (ii) Summer wolf kill interval in Scandinavia and Yellowstone National Park

To determine the effect of brown bears on wolf kill interval during summer, we evaluated whether brown bear presence (i) within wolf territories in SCA and (ii) at wolf-killed ungulates in YNP was an important predictor of kill interval. We used observations collected during 18 May–15 July in SCA (*N* = 12) and 1 May–31 July in YNP (*N* = 23). Inaccessibility of some clusters (2%; *N* = 103/4962) in YNP precluded a site search. This did not bias our estimate of YNP kill interval because our calculations only considered time periods during which all clusters were searched (except for unsearched clusters near the home site; see electronic supplementary material, appendix S1). Model variables in the SCA candidate model set included bear presence, Julian kill date (139–193), pack size (2–9), prey type, moose density (0.02–0.68) and distance to nearest road (0.008–1.16 km). Model variables in the YNP candidate model set included bear presence, Julian kill date (120–211), pack size (2–15), prey type, number of scavenged carcasses between kills (0–2) and distance to nearest road (0.03–16.61 km).

We conducted all analyses in R v. 3.0.1 [50] using general linear mixed models (GLMMs) using the ‘lmer’ function in the ‘lme4’ package v. 1.1–7 [51]. GLMMs can account for potential correlation between multiple observations taken on an individual wolf, from each pack, and within each year; pack identity and year were fitted *a priori* as crossed random effects in all models. Wolf identity was also included as a crossed random effect in YNP models. The kill interval in YNP was square-root-transformed to meet model assumptions. All models included a compound symmetric correlation structure, which assumed that all observations for each wolf, pack and year were, on average, equally correlated [52]. Model parameters were estimated using maximum likelihood.

We used Akaike information criterion (AIC) model selection [53] to test our three main predictions. The best-fit model had the lowest AIC score, which was adjusted for small sample size (AIC_c_). To determine the relative importance of our variables of interest, we examined whether they were retained in the top models (models with a ΔAIC_c_ < 2 [53]). The correlation coefficients between model variables were less than 0.6 in all model sets except for bear density and Julian date in the spring SCA analysis, which had a correlation coefficient of 0.7. We performed model averaging on models with ΔAIC_c_ < 2 to estimate *β* coefficients, standard errors and 95% confidence intervals (95% CI), using the ‘modavg’ function in the ‘AICcmodavg’ package v. 2.0–1 [54]. Population-averaged fitted values for graphs were calculated from best-fit models using the ‘PredictSE’ function in the ‘AICcmodavg’ package.

## 3. Results

### (a) Spring wolf kill interval in Scandinavia

**Figure 1.**
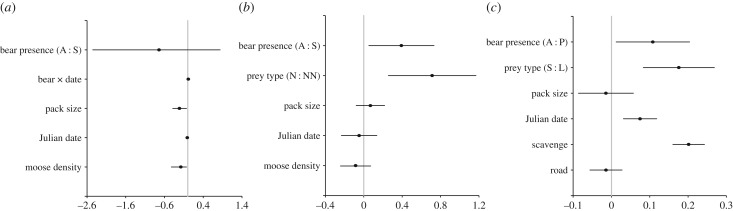
Parameter estimates from the top models predicting wolf kill interval (days between consecutive kills) for (*a*) spring and (*b*) summer in Scandinavia, and (*c*) summer in Yellowstone National Park (electronic supplementary material table S2). Model averaged estimates of *β*-coefficients, standard errors and 95% CIs were taken from the top models (ΔAIC_c_ < 2) for (*b*) and (*c*) (electronic supplementary material, table S3*b,c*). Interaction terms precluded model averaging, so estimates are reported from the top model for (*a*) (electronic supplementary material, table S3*a*). Continuous variables were centred and scaled in all models, and parameter estimates are on the square root scale for (*c*). The reference group for categorical variables is listed first in parentheses. Bear presence was defined as wolves being either allopatric (A) or sympatric (S) with brown bears in Scandinavia (*a,b*), or brown bears being absent (A) or present (P) at a wolf kill in Yellowstone National Park (*c*). Categorical variables for prey type included neonate (N) and non-neonate (NN) moose in Scandinavia (*b*), and small (S) and large (L) ungulate in Yellowstone National Park (*c*). ‘Bear × date’ refers to an interaction between bear presence and Julian date (*a*). Other independent variables included wolf pack size, Julian date of the kill (*a–c*), moose density (average number of moose harvested per square kilometre) (*a,b*), and number of scavenged carcasses between kills and distance (km) from the kill site to the nearest road (*c*).

**Figure 2.**
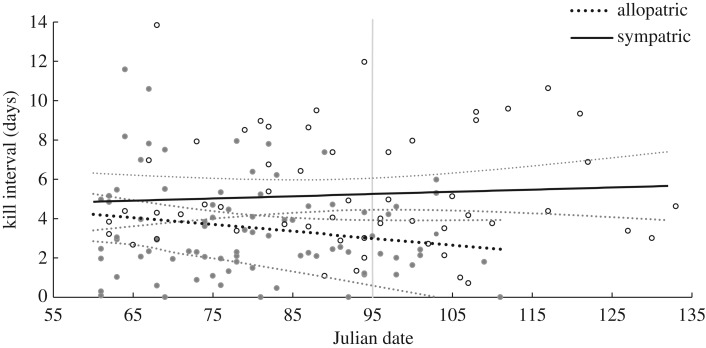
Effect of bear presence on the time interval (in days) between consecutive wolf-killed moose during the spring in wolf territories in Scandinavia. The lines indicate the population-averaged fitted values, with associated 95% CIs, from the best-fit GLMM of kill interval (electronic supplementary material, table S3*a*). Open and filled circles represent the data for wolf kills in sympatric and allopatric wolf–bear areas, respectively. The vertical grey line indicates the mean date of den emergence for male brown bears in Scandinavia (4 April).

We found no evidence that kill interval decreased across the spring bear emergence period for SCA wolves sympatric with brown bears. By contrast, all six top models of spring wolf kill interval in SCA (electronic supplementary material, table S2*a*) included a positive interaction between Julian date and bear presence (electronic supplementary material, table S3*a*; figure 1*a*; *N* = 140 observations across 12 packs and 11 years). This indicates that kill interval decreased across the spring emergence period for wolves that were allopatric, rather than sympatric, with bears (figure 2). The kill interval of sympatric wolves was effectively constant across the spring emergence period. Note, however, that the 95% CI for this interaction included 0 (electronic supplementary material, table S3*a*). Terms for pack size, moose density, prey type and snow depth also were retained in the top models (electronic supplementary material, table S2*a*). The best-fit model (electronic supplementary material, table S3*a*) indicated that time between wolf kills decreased with increasing moose density and pack size (figure 1*a*). Estimates from the top model that included a term for prey type and snow depth (electronic supplementary material, table S2*a*) suggested that kill interval increased when adult moose were killed compared with calves (*β* = 0.20; s.e. = 0.19) and decreased with increasing snow depth (*β* = −0.13; s.e. = 0.09), although the 95% CIs for these two estimates overlapped 0. Adult moose comprised 21% (*N* = 29/140) of all kills made by wolves during spring, and 24% (*N* = 20/84) and 16% (*N* = 9/56) of kills in allopatric and sympatric areas, respectively.

### (b) Summer wolf kill interval in Scandinavia and Yellowstone National Park

**Figure 3.**
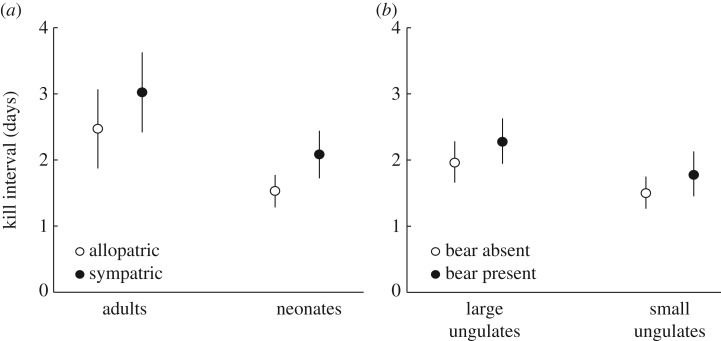
Effect of (*a*) bear presence in a wolf territory in Scandinavia and (*b*) bear presence at a wolf kill in Yellowstone National Park on the time interval (in days) between consecutive wolf kills in the summer. Open and closed circles are population-averaged fitted values with 95% CIs from the best-fit GLMMs of kill interval (electronic supplementary material, table S3*b,c*).

The variable for bear presence was retained in four of five top models of summer wolf kill interval in SCA (electronic supplementary material, table S2*b*; *N* = 157 observations across 10 packs and 6 years), and the 95% CI around its model averaged coefficient did not overlap 0, providing strong support for the positive direction of this effect (figure 1*b*). On average, the kill interval of sympatric packs was 12.1 ± 5.6 h longer than it was for allopatric packs (figure 3*a*). Mean (±s.e.) kill interval for all packs was 1.82 ± 1.33 days (43.68 ± 31.92 h), suggesting that bear presence in a wolf territory increased kill interval by about 28%. Terms for prey type, pack size, moose density and Julian date were included in the five top SCA models (electronic supplementary material, table S2*b*). Kill interval increased when wolves killed non-neonate moose compared to neonates (figure 1*b*; figure 3*a*). During the summer, non-neonate moose constituted 12% (*N* = 19/157) of all wolf kills in SCA, and comprised 9% (*N* = 10/106) and 18% (*N* = 9/51) of kills in allopatric and sympatric areas, respectively. In addition, kill interval decreased with moose density, and increased with pack size, although the 95% CIs for these estimates overlapped 0 (figure 1*b*).

Bear sign was found at 27% (*N* = 149/544) of the unique kills detected during summer in YNP. Although wolves killed more small ungulates (*N* = 312/544), bears used large ungulate kills more often; bear sign was found at 14% (*N* = 44/312) of small ungulate kills and at 45% (*N* = 105/232) of large ungulate kills. Bear presence was retained as a predictor of wolf kill interval in all three top models (electronic supplementary material, table S2*c*; *N* = 691 observations across 19 wolves, 10 packs and 8 years), and the 95% CI around the model averaged coefficient for bear presence did not overlap 0 (figure 1*c*). Kill interval increased when bears were present at kills (figure 3*b*); bear presence was associated with a 7.6 h increase in kill interval. The mean summer kill interval was 2.19 ± 1.99 days (52.7 ± 47.8 h), suggesting that bear presence increased kill interval by about 14%. Terms for prey type, scavenge events, Julian date, distance to nearest road and pack size were also retained in the top YNP models (electronic supplementary material, table S2*c*). Kill interval in YNP increased with the number of scavenge events, over the summer season, and when large ungulates were killed compared to small ungulates (figure 1*c*). Kill interval also decreased with pack size and distance to the nearest road, although the 95% CIs for these estimates overlapped 0 (figure 1*c*).

## 4. Discussion

Wolf kill interval was affected by several factors in both Scandinavia and Yellowstone, including prey type, wolf pack size and Julian date (figure 1). For example, wolf kill interval increased in both systems when wolves killed larger prey, as previously reported [18–19,55] (figure 1). Kill interval in Scandinavia also decreased as the abundance of wolves' primary prey, moose, increased, as previously demonstrated [55–57]. In Yellowstone, kill interval also increased as wolves scavenged more carcasses between kills. While these results highlight factors that are known to affect wolf kill interval [18–19,55–57], we also show a novel effect of brown bear presence.

Contrary to our hypotheses, the presence of brown bears resulted in wolves killing less frequently in both Scandinavia and Yellowstone. Wolf packs sympatric with brown bears in Scandinavia killed less often than allopatric packs in both spring and summer. In Yellowstone, where brown bear and wolf distributions overlapped, the presence of bears at wolf-killed ungulates was associated with wolves killing less often during summer. These results contradict the expectation that wolves kill more often where they coexist with brown bears, because the loss of food biomass from kleptoparasitism forces additional hunting to meet energetic demands [15,16].

The reason why brown bears are linked to increased wolf kill interval is not intuitive, but several mechanisms might cause this pattern. By definition, kill interval is the sum of time a predator spends handling (i.e. consuming) the first prey and searching for and killing the second. Interference competition can force a subordinate predator to prematurely abandon its kill, resulting in decreased handling time and, subsequently, shorter kill intervals (e.g. through kleptoparasitism [13,14]). Conversely, it is also possible that predators might realize greater fitness benefits from lingering at the usurped carcass, striving for occasional access, rather than prematurely abandoning it to make a new kill.

Hunting large ungulates is a difficult and dangerous task for wolves. Less than 25% of elk hunts in Yellowstone are successful [58,59] and wolves in Scandinavia succeed in killing moose about half the time (45–64% [40]). Hunts often necessitate a significant energy investment for wolves (e.g. chase distances can be long and hunting bouts can last hours [60]). Furthermore, wolves face a high risk of injury, or even death, when hunting large prey that can fight back [60,61]. Increased kill intervals could result, therefore, if wolves waited for occasions to feed on their kill while bears remained at the carcass, or if they waited for bears to leave, instead of abandoning their kills, as do Eurasian lynx (*Lynx lynx*) [13] and mountain lions (*Puma concolor*) [14,62]. This would be expected with larger prey, where longer time spent at the kill site could increase the potential for interactions and where more biomass is likely to remain once the kill has been relinquished by the bear.

Alternatively, exploitative competition may increase kill interval if greater time investment or superior search efficiency by one predator diminishes the supply of a shared prey, thus leading to an increase in search time for a second predator and lengthening kill interval [63]. In many systems where they occur, brown bears are the most significant predator of neonate ungulates [64]. In Scandinavia, bears accounted for approximately 90% of total neonate moose mortality when allopatric with the wolf population [65]. In Yellowstone, predators accounted for 94% of all neonate elk mortality within the first 30 days of life; brown and black bears accounted for 69% of those deaths, whereas wolves accounted for 12% [37]. Therefore, successive depletion of neonate prey by both brown bears and wolves could have caused increased search times and, subsequently, an increased wolf kill interval, during summer in both systems.

It is also possible that facilitation, rather than competition, from brown bears increased wolf kill interval. Frequent predation by bears could increase scavenging opportunities for wolves, thereby lengthening wolf kill interval. However, there is little evidence for this mechanism in Scandinavia or Yellowstone. Although bears are important predators of neonates during early summer in both systems [37,65], neonates are small and quickly consumed, with little or no biomass remaining for scavengers. To date, there have been no confirmed cases of adult wolves using neonate bear kills in Scandinavia [66]. Furthermore, brown bears in Scandinavia and Yellowstone rarely kill adult ungulates [28,67], whose carcasses would be more likely to retain useable biomass.

During spring in Scandinavia, it is more likely that interference competition caused increased kill intervals, as wolves and bears do not predate on the same resource (i.e. neonate moose) at this time of year, as compared with early summer. However, neonate moose represented the majority of wolf kills made by both sympatric (82%) and allopatric packs (91%) during the summer in Scandinavia. Although we controlled for variation in moose density, we were unable to account for brown bear-induced changes to neonate prey density during summer, which could have explained the observed difference in kill interval between sympatric and allopatric packs in summer. In Yellowstone, small ungulate prey, including neonates, accounted for approximately 57% of the 544 kills, although 70% of the detected bear sign was at large ungulate kills. Whereas wolves in Yellowstone kill neonate ungulates frequently during summer, large ungulates supply the majority of acquired biomass [19]. Thus, it is possible that increased summer wolf kill interval was the result of multiple mechanisms: bears reducing densities of neonate ungulates (i.e. exploitative competition) and wolves loitering at larger, usurped kills (i.e. interference competition). Future research should tease apart the relative role of interference and exploitative competition between apex predators in driving seasonal predation patterns in different ecosystems.

Although we used two large datasets at a transcontinental scale to improve our understanding of competition between two apex predators, there were some limitations in our study. For instance, bear ‘presence’ was differentially defined in Scandinavia and Yellowstone, and kill interval was calculated at different levels (i.e. pack versus individual) in the two systems. However, our results were consistent across seasonal and transcontinental scales; bear presence increased wolf kill interval (i.e. decreased kill rate) in both Scandinavia and Yellowstone during spring and summer. These findings suggest that competition between brown bears and wolves actually extended the kill interval of wolves in Scandinavia (figures 1*a,b*,2 and 3*a*) and Yellowstone (figures 1*c* and 3*b*).

Our results challenge the conventional view that brown bears do not affect the distribution, survival or reproduction of wolves [68]. For example, extended wolf kill intervals in areas sympatric with bears may help explain why wolf pair establishment in Scandinavia was negatively related to bear density, among other intraspecific and environmental factors [44]. Although the outcome of interactions between bears and wolves at carcasses varies, bears often dominate, limiting wolves' access to food [15,16,36]. Furthermore, our findings suggest that wolves do not hunt more often to compensate for the loss of food to brown bears. In combination, this implies that bears might negatively affect the food intake of wolves, such that wolf populations that are sympatric with brown bears might suffer fitness consequences. Determining the energetic costs of these interactions (e.g. food biomass lost and energy expended by wolves) and linking them to predator population dynamics will ultimately help us understand the costs of sympatry among apex predator populations.

Although bears seemingly caused fewer prey to be killed by wolves, it is difficult to ascertain how this ultimately affected the cumulative predation rate of the respective ungulate populations, as we only examined wolf predation. Whereas predation by brown bears on neonates is well understood, and can be additive to other predator-induced mortality [64], our results suggest the possibility that the total impact of wolves and brown bears on non-neonate prey may be less than the sum of their individual impacts. If so, the outcome of interactions between wolves and bears may mitigate, rather than exacerbate, the influence of these carnivores on ungulate population dynamics.

Our results provide new information about the consequences of competition between apex predators that is relevant to understanding how large predator diversity affects trophic interactions in natural systems. Interspecific interactions between apex predators can either relax or strengthen their cumulative effect on prey populations and overall ecosystem functioning [9,10]. Ignoring such interactions may result in underestimating the effect that interspecific competition between predators can have on predator populations, as well as overestimating the impact of multiple predators on prey population dynamics.

## Supplementary Material

Appendix S1

## Supplementary Material

Table S1

## Supplementary Material

Table S2

## Supplementary Material

Table S3

## Data accessibility

Data available from the Dryad Digital Repository: http://dx.doi.org/10.5061/dryad.18nh4 [69].
