# Supplementary material for: Competition between apex predators? Brown bears decrease wolf kill rate on two continents
Source: Proc Biol Sci. 2017 Feb 8;284(1848):20162368. doi: 10.1098/rspb.2016.2368 (PMC5310606; doi:10.1098/rspb.2016.2368)
Supplement: Appendix S1 [file rspb20162368supp1.docx]

**Appendix S1. Cluster searches and carcass cause of death**

Field crews searched for carcasses within a 100 m radius of all ‘clustered’ GPS points in Scandinavia, and within a 400 m^2^ area of all clustered GPS points in Yellowstone. Clusters were not visited until the wolves had moved away from the site, and visits were generally made 2-4 days after the cluster originated. From May onward in both systems, clusters near the home site (i.e., within 2 km (SCA) and 1 km (YNP) of where pups were raised) were not searched immediately to not disrupt wolf denning behavior. In Scandinavia, these clusters were searched after the home site was abandoned in 3 of the 4 territories sympatric with bears, and no additional carcasses were found. In Yellowstone, most clusters near home sites were searched post wolf abandonment (4% were never searched; N=196/4962). Bias in kill rate due to unsearched clusters was likely minimal as most clusters near home sites were not associated with carcass visits. Instead, these clusters were typically generated by i) non-consecutive, but spatially overlapping, GPS points associated with travel to and from the home site or ii) GPS points that occurred mid-day which were very near the home site; the spatial and temporal characteristics of these clusters were suggestive of bed sites [1].

Upon carcass detection, we determined cause of death by searching each carcass and carcass site for signs of predation such as blood trails, subcutaneous hemorrhaging, canine punctures, vegetation disturbance, scat, tracks, and hair. We designated predator species using scat, tracks, and hair, and predator-specific patterns of consumption (e.g., location of canine punctures, separation of rumen from the carcass, disarticulation of the ungulate skeleton, or burial of the carcass). Furthermore, we classified carcasses as wolf-killed if its state of decomposition and suspected time of death matched spatially and temporally with GPS positions from collared wolves. All carcasses were classified into 3 categories: 1) definite wolf-killed prey, 2) probable wolf-killed prey, and 3) died from other causes (e.g., other predators, other natural causes, etc.). We assumed wolves were scavenging when they visited a carcass that had not been killed by their pack (i.e., time of death matched spatially and temporally with GPS positions from wolves in a different pack, or the ungulate died from other causes).

Brown bears rarely prey on adult ungulates (i.e., adult moose in Scandinavia and adult elk, bison, or moose ≥11 months in Yellowstone) in either system [2, 3]. Evidence of bear presence at a carcass included bear scat, tracks, or hair, or characteristic signs of bear consumption (e.g., twisted remains, and crushed large bones such as femur and skull, and carcass covered with soil and vegetation). We assumed that neonate carcasses located at cluster positions (i.e., > 2 GPS positions) were killed by wolves, unless we found evidence that the kill was made by another predator. Neonate prey are consumed quickly by bears and wolves [4], and we surmised that if wolves spent enough time at a neonate carcass to create a cluster, this represented a kill. In the areas where bears and wolves overlapped in Scandinavia, we detected bear sign at, or near, 21% of 33 neonate moose kills. Note that bear sign was recorded for 3 out of 4 study packs that overlapped bear territory. In Yellowstone, bear sign was found at, or near, 14% of 312 small ungulate wolf-kills.

**REFERENCES**

1. Metz M.C., Vucetich J.A., Smith D.W., Stahler D.R., Peterson R.O. 2011 Effect of sociality and season on gray wolf (*Canis lupus*) foraging behavior: implications for estimating summer kill rate. *PloS one* **6**(3), e17332. (doi:10.1371/journal.pone.0017332).

2. Evans S., Mech D., White P.J., Sargeant G. 2006 Survival of adult female elk in Yellowstone following wolf restoration. *Journal of Wildlife Management* **70**(5), 1372-1378.

3. Dahle B., Wallin K., Cederlund G., Persson I.L., Selvaag L., Swenson J.E. 2013 Predation on adult moose *Alces alces* by European brown bears *Ursus arctos*. *Wildlife Biology* **19**(2), 165-169.

4. Barber-Meyer S.M., Mech L.D., White P.J. 2008 Elk calf survival and mortality following wolf restoration to Yellowstone National Park. *Wildlife Monographs* **169**, 1-30. (doi:10.2193/2008-004).
