# Supplementary material for: Competition between apex predators? Brown bears decrease wolf kill rate on two continents
Source: Proc Biol Sci. 2017 Feb 8;284(1848):20162368. doi: 10.1098/rspb.2016.2368 (PMC5310606; doi:10.1098/rspb.2016.2368)
Supplement: Table S1 [file rspb20162368supp2.docx]

**Table S1.** Summary of predation studies carried out in spring (a) and summer (b) in Scandinavia and summer in Yellowstone National Park (c). The sex (M/F) of the followed wolf is indicated at the end of each wolf ID number.

| **(a) Scandinavia – Spring** | |  |  |  |
| --- | --- | --- | --- | --- |
| **Wolf Territory** | **Wolf Followed** | **Study Period** | **Duration of Study (Days)** | **Sympatric with Bears** |
| Gråfjell | M0109M | 12/11/01 - 04/21/02 | 132 | No |
| Gråfjell | M0109M | 02/17/03 - 04/20/03 | 63 | No |
| Tyngsjö | M0204F | 01/31/02 - 04/24/02 | 84 | No |
| Bograngen | M0009M | 02/17/03 - 04/20/03 | 63 | No |
| Nyskoga | M0007M | 02/13/04 - 03/16/04 | 33 | No |
| Djurskog | M0306M | 02/01/04 - 03/28/04 | 56 | No |
| Jangen | M0404M | 02/02/04 - 04/01/04 | 60 | No |
| Gräsmark | M0611M / M0610F | 02/18/07 - 04/09/07 | 50 | No |
| Kloten | M0910M | 02/11/08 - 03/31/08 | 50 | No |
| Fulufjället | M0904M | 02/15/09 - 04/08/09 | 52 | Yes |
| Fulufjället | M0904M | 04/01/10 - 06/01/10 | 61 | Yes |
| Tenskog | M1002M | 02/13/10 - 04/11/10 | 57 | Yes |
| Tenskog | M1003M | 03/14/11 - 05/16/11 | 63 | Yes |
| Tandsjön | M1103M | 02/20/12 - 05/14/12 | 84 | Yes |
| Kukumäki | M1302M | 02/25/13 - 04/28/13 | 62 | Yes |
| Tandsjön | M1103M | 03/19/14 - 04/25/14 | 37 | Yes |
| Kukumäki | M1302M | 03/03/14 - 04/25/14 | 53 | Yes |
| Kukumäki | M1301F | 03/04/15 - 04/24/15 | 51 | Yes |
|  |  |  |  |  |
| **(b) Scandinavia – Summer** | |  |  |  |
| **Wolf Territory** | **Wolf Followed** | **Study Period** | **Duration of Study (Days)** | **Sympatric with Bears** |
| Nyskoga | M0007M | 06/02/03 - 06/10/03 | 8 | No |
| Gråfjell | M0109M | 06/02/03 - 07/14/03 | 42 | No |
| Bograngen | M0009M | 06/02/03 - 07/14/03 | 42 | No |
| Halgån | M0206F | 06/21/03 - 07/14/03 | 23 | No |
| Djurskog | M0306M | 06/21/04 - 07/12/04 | 21 | No |
| Koppang | M0402M | 06/14/04 - 07/05/04 | 21 | No |
| Gråfjell | M0109M | 06/14/04 - 07/05/04 | 21 | No |
| Kloten | M0918M | 06/13/09 - 07/11/09 | 28 | No |
| Tenskog | M1002M | 05/30/11 - 06/26/11 | 27 | Yes |
| Tandsjön | M1103M | 05/19/14 - 06/21/14 | 33 | Yes |
| Kukumäki | M1302M | 05/19/14 - 06/22/14 | 34 | Yes |
| Kukumäki | M1301F | 05/18/15 - 06/29/15 | 42 | Yes |
|  |  |  |  |  |
|  |  |  |  |  |
| **(c) Yellowstone – Summer** | |  |  |  |
| **Wolf Territory** | **Wolf Followed** | **Study Period** | **Duration of Study (Days)** |  |
| Leopold | 624F | 05/01/08 - 08/01/08 | 92 |  |
| Leopold | 625F | 05/01/08 - 08/01/08 | 92 |  |
| Oxbow Creek | 626F | 05/01/08 - 08/01/08 | 92 |  |
| Oxbow Creek | 627M | 05/01/08 - 08/01/08 | 92 |  |
| Blacktail | 692F | 05/01/09 - 08/01/09 | 92 |  |
| Blacktail | 693F | 05/01/09 - 08/01/09 | 92 |  |
| Everts | 684M | 05/01/09 - 08/01/09 | 92 |  |
| Everts | 685M | 05/01/09 - 08/01/09 | 92 |  |
| Blacktail | 642F | 05/01/10 - 08/01/10 | 92 |  |
| Blacktail | 752F | 05/01/10 - 08/01/10 | 92 |  |
| Agate Creek | 775M | 06/01/11 - 06/30/11 | 29 |  |
| Blacktail | 777M | 05/01/11 - 08/01/11 | 92 |  |
| Blacktail | 777M | 05/01/12 - 05/28/12 | 27 |  |
| Blacktail | 829F | 05/01/12 - 08/01/12 | 92 |  |
| Junction Butte | 777M | 05/31/12 - 08/01/12 | 62 |  |
| 8 Mile | SW763M | 05/01/13 - 08/01/13 | 92 |  |
| 889F/890M Group | 889F | 05/01/13 - 06/27/13 | 57 |  |
| 889F/890M Group | 890M | 05/01/13 - 08/01/13 | 92 |  |
| 911M Group | 911M | 05/13/14 - 07/01/14 | 49 |  |
| Junction Butte | 890M | 05/01/14 - 07/01/14 | 61 |  |
| Junction Butte | 907F | 05/01/14 - 07/01/14 | 61 |  |
| Junction Butte | 911M | 05/01/14 - 05/13/14 | 12 |  |
| Prospect Peak | 964M | 05/01/15 - 08/01/15 | 92 |  |
