# Supplementary material for: Competition between apex predators? Brown bears decrease wolf kill rate on two continents
Source: Proc Biol Sci. 2017 Feb 8;284(1848):20162368. doi: 10.1098/rspb.2016.2368 (PMC5310606; doi:10.1098/rspb.2016.2368)
Supplement: Table S2 [file rspb20162368supp3.docx]

**Table S2.** *A priori* GLMM model sets predicting wolf kill interval (days between consecutive kills) during spring (a) and summer (b) in Scandinavia and summer in Yellowstone National Park (c). The null models contain the intercept and crossed random effects for pack ID and year (a-b) and pack ID, wolf ID and year (c). Log-likelihood (LogLike), number of parameters (K), AIC_c_, differences in AIC_c_ compared to the best scoring model (ΔAIC_c_), and AIC_c_ weights (Wi) are given for each model. The top models (ΔAIC < 2) are highlighted in bold. Bear presence was defined as wolves being either allopatric or sympatric with brown bears in Scandinavia (a-b), or brown bears being absent or present at a wolf kill in Yellowstone National Park (c). Continuous variables were centered and scaled in all models. Categorical variables for prey type were: adult or calf moose (a), neonate and non-neonate moose (b), and small and large ungulate (c). Other independent variables included wolf pack size, Julian date of the kill, snow depth (m) in the territory at kill date, moose density (average number of moose harvested/km^2^), the number of carcasses scavenged between kills, and distance (km) from the kill site to the nearest road. ‘Bear presence x Julian date’ refers to an interaction between the two variables (a).

| **(a) Scandinavia - Spring** |  |  |  |  |  |
| --- | --- | --- | --- | --- | --- |
|  |  |  |  |  |  |
| Model Set | LogLike | K | AICc | ∆AICc | Wi |
| **Bear Presence x Julian Date + Moose Density + Pack Size** | **-183.90** | **9** | **387.19** | **0.00** | **0.12** |
| **Bear Presence x Julian Date + Pack Size + Moose Density + Snow Depth** | **-182.86** | **10** | **387.43** | **0.24** | **0.11** |
| **Bear Presence x Julian Date + Pack Size** | **-185.23** | **8** | **387.56** | **0.37** | **0.10** |
| **Bear Presence x Julian Date + Prey Type + Pack Size + Moose Density** | **-183.39** | **10** | **388.48** | **1.29** | **0.07** |
| **Bear Presence x Julian Date + Prey Type + Pack Size** | **-184.63** | **9** | **388.64** | **1.45** | **0.06** |
| **Bear Presence x Julian Date + Pack Size + Snow Depth** | **-184.68** | **9** | **388.75** | **1.56** | **0.06** |
| Bear Presence x Julian Date | -187.17 | 7 | 389.19 | 2.00 | 0.05 |
| Bear Presence x Julian Date + Prey Type + Pack Size + Moose Density + Snow Depth | -182.64 | 11 | 389.34 | 2.14 | 0.04 |
| Bear Presence x Julian Date + Snow Depth | -186.18 | 8 | 389.45 | 2.26 | 0.04 |
| Bear Presence x Julian Date + Prey Type | -186.53 | 8 | 390.17 | 2.98 | 0.03 |
| Bear Presence x Julian Date + Prey Type + Pack Size + Moose Density + Road | -183.07 | 11 | 390.20 | 3.00 | 0.03 |
| Bear Presence x Julian Date + Moose Density + Snow Depth | -185.41 | 9 | 390.20 | 3.01 | 0.03 |
| Bear Presence x Julian Date + Prey Type + Pack Size + Snow Depth | -184.28 | 10 | 390.27 | 3.08 | 0.03 |
| Bear Presence x Julian Date + Prey Type + Pack Size + Road | -184.36 | 10 | 390.42 | 3.23 | 0.02 |
| Bear Presence x Julian Date + Road | -186.73 | 8 | 390.57 | 3.38 | 0.02 |
| Bear Presence x Julian Date + Moose Density | -186.86 | 8 | 390.81 | 3.62 | 0.02 |
| Bear Presence x Julian Date + Prey Type + Pack Size + Moose Density + Snow Depth + Road | -182.24 | 12 | 390.93 | 3.74 | 0.02 |
| Bear Presence x Julian Date + Prey Type + Snow Depth | -185.81 | 9 | 391.00 | 3.81 | 0.02 |
| Moose Density + Pack Size | -189.19 | 6 | 391.02 | 3.82 | 0.02 |
| Moose Density + Pack Size + Snow Depth | -188.11 | 7 | 391.07 | 3.88 | 0.02 |
| Bear Presence x Julian Date + Prey Type + Road | -186.20 | 9 | 391.79 | 4.60 | 0.01 |
| Bear Presence x Julian Date + Moose Density + Prey Type | -186.26 | 9 | 391.90 | 4.71 | 0.01 |
| Bear Presence x Julian Date + Prey Type + Moose Density + Snow Depth | -185.16 | 10 | 392.03 | 4.84 | 0.01 |
| Prey Type + Pack Size + Moose Density | -189.04 | 7 | 392.92 | 5.73 | 0.01 |
| Moose Density + Snow Depth | -190.25 | 6 | 393.13 | 5.94 | 0.01 |
| Prey Type + Pack Size + Moose Density + Snow Depth | -188.03 | 8 | 393.17 | 5.97 | 0.01 |
| Moose Density | -191.48 | 5 | 393.41 | 6.22 | 0.01 |
| Bear Presence x Julian Date + Prey Type + Moose Density + Road | -185.90 | 10 | 393.50 | 6.31 | 0.01 |
| Julian Date + Moose Density + Snow Depth | -189.72 | 7 | 394.28 | 7.09 | 0.00 |
| Prey Type + Pack Size + Moose Density + Road | -188.78 | 8 | 394.67 | 7.48 | 0.00 |
| Prey Type + Pack Size + Moose Density + Snow Depth + Road | -187.71 | 9 | 394.81 | 7.62 | 0.00 |
| Null (Intercept Only) | -193.31 | 4 | 394.92 | 7.72 | 0.00 |
| Prey Type + Moose Density + Snow Depth | -190.13 | 7 | 395.12 | 7.92 | 0.00 |
| Moose Density + Prey Type | -191.26 | 6 | 395.15 | 7.96 | 0.00 |
| Julian Date + Prey Type + Pack Size + Moose Density | -189.03 | 8 | 395.16 | 7.97 | 0.00 |
| Julian Date + Prey Type + Pack Size + Moose Density + Snow Depth | -187.91 | 9 | 395.21 | 8.02 | 0.00 |
| Julian Date + Moose Density | -191.44 | 6 | 395.51 | 8.32 | 0.00 |
| Pack Size | -192.59 | 5 | 395.63 | 8.44 | 0.00 |
| Julian Date + Prey Type + Moose Density + Snow Depth | -189.66 | 8 | 396.41 | 9.22 | 0.00 |
| Prey Type | -193.01 | 5 | 396.47 | 9.28 | 0.00 |
| Snow Depth | -193.02 | 5 | 396.50 | 9.30 | 0.00 |
| Julian Date + Prey Type + Pack Size + Moose Density + Road | -188.78 | 9 | 396.95 | 9.75 | 0.00 |
| Julian Date | -193.29 | 5 | 397.03 | 9.83 | 0.00 |
| Prey Type + Pack Size | -192.32 | 6 | 397.28 | 10.09 | 0.00 |
| Julian Date + Moose Density + Prey Type | -191.22 | 7 | 397.30 | 10.10 | 0.00 |
| Pack Size + Snow Depth | -192.41 | 6 | 397.45 | 10.25 | 0.00 |
| Prey Type + Road | -192.70 | 6 | 398.03 | 10.84 | 0.00 |
| Prey Type + Snow Depth | -192.82 | 6 | 398.26 | 11.07 | 0.00 |
| Julian Date + Snow Depth | -192.85 | 6 | 398.33 | 11.13 | 0.00 |
| Julian Date + Prey Type | -192.98 | 6 | 398.59 | 11.40 | 0.00 |
| Prey Type + Pack Size + Road | -192.04 | 7 | 398.93 | 11.74 | 0.00 |
| Prey Type + Pack Size + Snow Depth | -192.21 | 7 | 399.26 | 12.07 | 0.00 |
| Julian Date + Prey Type + Pack Size | -192.32 | 7 | 399.50 | 12.30 | 0.00 |
| Julian Date + Pack Size + Snow Depth | -192.36 | 7 | 399.57 | 12.38 | 0.00 |
| Julian Date + Prey Type + Snow Depth | -192.65 | 7 | 400.15 | 12.96 | 0.00 |
| Julian Date + Prey Type + Pack Size + Road | -192.04 | 8 | 401.18 | 13.98 | 0.00 |
| Julian Date + Prey Type + Pack Size + Snow Depth | -192.17 | 8 | 401.43 | 14.24 | 0.00 |
|  |  |  |  |  |  |
| **(b) Scandinavia - Summer** |  |  |  |  |  |
|  |  |  |  |  |  |
| Model Set | LogLike | K | AICc | ∆AICc | Wi |
| **Bear Presence + Prey Type** | **-213.77** | **6** | **440.10** | **0.00** | **0.15** |
| **Bear Presence + Moose Density + Prey Type** | **-213.20** | **7** | **441.16** | **1.06** | **0.09** |
| **Bear Presence + Prey Type + Pack Size** | **-213.35** | **7** | **441.46** | **1.36** | **0.08** |
| **Prey Type** | **-215.75** | **5** | **441.89** | **1.79** | **0.06** |
| **Bear Presence + Prey Type + Julian Date** | **-213.64** | **7** | **442.02** | **1.93** | **0.06** |
| Prey Type + Road | -214.93 | 6 | 442.43 | 2.33 | 0.05 |
| Prey Type + Moose Density + Julian Date | -213.88 | 7 | 442.51 | 2.42 | 0.05 |
| Moose Density + Prey Type | -215.01 | 6 | 442.59 | 2.49 | 0.04 |
| Bear Presence + Moose Density + Prey Type + Road | -212.81 | 8 | 442.60 | 2.50 | 0.04 |
| Prey Type + Julian Date | -215.08 | 6 | 442.72 | 2.63 | 0.04 |
| Bear Presence + Prey Type + Pack Size + Road | -212.90 | 8 | 442.78 | 2.68 | 0.04 |
| Prey Type + Moose Density + Road | -214.06 | 7 | 442.86 | 2.77 | 0.04 |
| Bear Presence + Moose Density + Prey Type + Julian Date | -213.01 | 8 | 442.99 | 2.90 | 0.04 |
| Bear Presence + Prey Type + Pack Size + Moose Density | -213.10 | 8 | 443.17 | 3.07 | 0.03 |
| Bear Presence + Prey Type + Pack Size + Julian Date | -213.28 | 8 | 443.52 | 3.43 | 0.03 |
| Prey Type + Pack Size | -215.69 | 6 | 443.94 | 3.84 | 0.02 |
| Prey Type + Pack Size + Road | -214.73 | 7 | 444.21 | 4.12 | 0.02 |
| Bear Presence + Moose Density + Prey Type + Pack Size + Road | -212.66 | 9 | 444.55 | 4.45 | 0.02 |
| Prey Type + Pack Size + Julian Date | -214.95 | 7 | 444.65 | 4.56 | 0.02 |
| Prey Type + Pack Size + Moose Density + Julian Date | -213.88 | 8 | 444.73 | 4.64 | 0.02 |
| Prey Type + Pack Size + Moose Density | -215.01 | 7 | 444.77 | 4.67 | 0.01 |
| Prey Type + Pack Size + Moose Density + Road | -214.01 | 8 | 444.99 | 4.90 | 0.01 |
| Bear Presence + Moose Density + Prey Type + Pack Size + Julian Date | -212.95 | 9 | 445.13 | 5.03 | 0.01 |
| Prey Type + Pack Size + Moose Density + Road + Julian Date | -213.11 | 9 | 445.44 | 5.35 | 0.01 |
| Bear Presence + Prey Type + Pack Size + Moose Density + Road + Julian Date | -212.54 | 10 | 446.58 | 6.48 | 0.01 |
| Bear Presence | -218.27 | 5 | 446.94 | 6.84 | 0.00 |
| Bear Presence + Moose Density | -217.83 | 6 | 448.23 | 8.13 | 0.00 |
| Bear Presence + Road | -217.92 | 6 | 448.40 | 8.30 | 0.00 |
| Bear Presence + Pack Size | -218.06 | 6 | 448.67 | 8.57 | 0.00 |
| Null (Intercept Only) | -220.40 | 4 | 449.07 | 8.97 | 0.00 |
| Moose Density | -219.89 | 5 | 450.18 | 10.08 | 0.00 |
| Bear Presence + Pack Size + Julian Date | -217.73 | 7 | 450.20 | 10.11 | 0.00 |
| Moose Density + Road | -219.03 | 6 | 450.63 | 10.53 | 0.00 |
| Moose Density + Pack Size + Julian Date | -218.22 | 7 | 451.19 | 11.09 | 0.00 |
| Pack Size | -220.40 | 5 | 451.20 | 11.10 | 0.00 |
| Bear Presence + Moose Density + Pack Size + Julian Date | -217.35 | 8 | 451.67 | 11.57 | 0.00 |
| Bear Presence + Moose Density + Pack Size + Road | -217.41 | 8 | 451.80 | 11.70 | 0.00 |
| Moose Density + Pack Size | -219.77 | 6 | 452.11 | 12.01 | 0.00 |
| Moose Density + Pack Size + Road | -219.01 | 7 | 452.78 | 12.68 | 0.00 |
|  |  |  |  |  |  |
| **(c) Yellowstone - Summer** |  |  |  |  |  |
|  |  |  |  |  |  |
| Model Set | LL | K | AICc | Delta_AICc | AICcWt |
| **Bear Presence + Prey Type + Scavenge + Julian Date** | **-555.63** | **9** | **1129.52** | **0.00** | **0.40** |
| **Bear Presence + Prey Type + Scavenge + Julian Date + Road** | **-555.42** | **10** | **1131.16** | **1.64** | **0.18** |
| **Bear Presence + Prey Type + Pack Size + Scavenge + Julian Date** | **-555.56** | **10** | **1131.45** | **1.93** | **0.15** |
| Prey Type + Scavenge + Julian Date | -558.07 | 8 | 1132.35 | 2.83 | 0.10 |
| Bear Presence + Prey Type + Pack Size + Scavenge + Julian Date + Road | -555.36 | 11 | 1133.10 | 3.58 | 0.07 |
| Prey Type + Scavenge + Julian Date + Road | -557.83 | 9 | 1133.92 | 4.40 | 0.04 |
| Prey Type + Pack Size + Scavenge + Julian Date | -557.97 | 9 | 1134.20 | 4.68 | 0.04 |
| Prey Type + Pack Size + Scavenge + Julian Date + Road | -557.73 | 10 | 1135.79 | 6.26 | 0.02 |
| Bear Presence + Prey Type + Scavenge | -561.25 | 8 | 1138.72 | 9.19 | 0.00 |
| Bear Presence + Prey Type + Pack Size + Scavenge | -561.03 | 9 | 1140.33 | 10.81 | 0.00 |
| Bear Presence + Prey Type + Scavenge + Road | -561.14 | 9 | 1140.53 | 11.01 | 0.00 |
| Bear Presence + Prey Type + Pack Size + Scavenge + Road | -560.92 | 10 | 1142.17 | 12.64 | 0.00 |
| Prey Type + Scavenge | -564.33 | 7 | 1142.82 | 13.30 | 0.00 |
| Bear Presence + Pack Size + Scavenge + Julian Date | -562.44 | 9 | 1143.14 | 13.62 | 0.00 |
| Bear Presence + Scavenge | -564.84 | 7 | 1143.84 | 14.32 | 0.00 |
| Prey Type + Pack Size + Scavenge | -564.04 | 8 | 1144.28 | 14.76 | 0.00 |
| Prey Type + Scavenge + Road | -564.19 | 8 | 1144.59 | 15.07 | 0.00 |
| Bear Presence + Pack Size + Scavenge + Julian Date + Road | -562.19 | 10 | 1144.70 | 15.17 | 0.00 |
| Bear Presence + Pack Size + Scavenge | -564.77 | 8 | 1145.74 | 16.22 | 0.00 |
| Prey Type + Pack Size + Scavenge + Road | -563.91 | 9 | 1146.08 | 16.56 | 0.00 |
| Bear Presence + Pack Size + Scavenge + Road | -564.60 | 9 | 1147.47 | 17.94 | 0.00 |
| Pack Size + Scavenge + Julian Date | -569.10 | 8 | 1154.41 | 24.89 | 0.00 |
| Scavenge | -571.17 | 6 | 1154.46 | 24.94 | 0.00 |
| Pack Size + Scavenge + Julian Date + Road | -568.78 | 9 | 1155.82 | 26.30 | 0.00 |
| Pack Size + Scavenge | -571.09 | 7 | 1156.34 | 26.82 | 0.00 |
| Pack Size + Scavenge + Road | -570.86 | 8 | 1157.93 | 28.41 | 0.00 |
| Bear Presence + Prey Type + Julian Date | -598.52 | 8 | 1213.25 | 83.73 | 0.00 |
| Bear Presence + Prey Type + Pack Size + Julian Date | -597.84 | 9 | 1213.94 | 84.42 | 0.00 |
| Prey Type + Julian Date | -600.41 | 7 | 1214.98 | 85.46 | 0.00 |
| Bear Presence + Prey Type | -600.59 | 7 | 1215.35 | 85.82 | 0.00 |
| Prey Type + Pack Size + Julian Date | -599.66 | 8 | 1215.53 | 86.01 | 0.00 |
| Bear Presence + Prey Type + Pack Size | -599.68 | 8 | 1215.57 | 86.05 | 0.00 |
| Bear Presence + Prey Type + Pack Size + Julian Date + Road | -597.68 | 10 | 1215.69 | 86.17 | 0.00 |
| Bear Presence + Prey Type + Road | -600.49 | 8 | 1217.18 | 87.66 | 0.00 |
| Prey Type + Pack Size + Julian Date + Road | -599.49 | 9 | 1217.24 | 87.72 | 0.00 |
| Bear Presence + Prey Type + Pack Size + Road | -599.57 | 9 | 1217.41 | 87.89 | 0.00 |
| Prey Type | -602.84 | 6 | 1217.81 | 88.28 | 0.00 |
| Prey Type + Pack Size | -601.83 | 7 | 1217.83 | 88.31 | 0.00 |
| Prey Type + Road | -602.72 | 7 | 1219.61 | 90.09 | 0.00 |
| Prey Type + Pack Size + Road | -601.72 | 8 | 1219.64 | 90.12 | 0.00 |
| Bear Presence | -605.97 | 6 | 1224.07 | 94.55 | 0.00 |
| Bear Presence + Pack Size | -605.50 | 7 | 1225.16 | 95.64 | 0.00 |
| Bear Presence + Julian Date | -605.71 | 7 | 1225.59 | 96.06 | 0.00 |
| Bear Presence + Pack Size + Julian Date | -605.32 | 8 | 1226.85 | 97.32 | 0.00 |
| Bear Presence + Pack Size + Road | -605.33 | 8 | 1226.88 | 97.36 | 0.00 |
| Julian Date | -611.49 | 5 | 1233.07 | 103.55 | 0.00 |
| Null | -611.67 | 5 | 1233.42 | 103.90 | 0.00 |
| Pack Size | -611.20 | 6 | 1234.52 | 104.99 | 0.00 |
| Pack Size + Road | -610.99 | 7 | 1236.14 | 106.62 | 0.00 |
| Pack Size + Julian Date | -611.09 | 7 | 1236.34 | 106.81 | 0.00 |
