# Supplementary material for: Competition between apex predators? Brown bears decrease wolf kill rate on two continents
Source: Proc Biol Sci. 2017 Feb 8;284(1848):20162368. doi: 10.1098/rspb.2016.2368 (PMC5310606; doi:10.1098/rspb.2016.2368)
Supplement: Table S3 [file rspb20162368supp4.docx]

**Table S3.** Parameter estimates from the top models predicting wolf kill interval (days between consecutive kills) for spring (a) and summer (b) in Scandinavia and summer in Yellowstone National Park (c) (Table S2). Model-averaged estimates of β-coefficients, SEs and 95% confidence intervals were taken from the top models (ΔAIC_c_ < 2) for (b) and (c). Interaction terms precluded model averaging, so estimates are reported from the top model for (a). Continuous variables were centered and scaled in all models, and parameter estimates are on the square root scale for (c). The reference group for categorical variables is listed first in parentheses. Bear presence was defined as wolves being either allopatric (A) or sympatric (S) with brown bears in Scandinavia (a-b), or brown bears being absent (A) or present (P) at a wolf kill in Yellowstone National Park (c). Categorical variables for prey type included neonate (N) and non-neonate (NN) moose in Scandinavia (b), and small (S) and large (L) ungulate in Yellowstone National Park (c). ‘Bear presence x Julian date’ refers to an interaction between the two variables (a). Other independent variables included wolf pack size, Julian date of the kill (a-c), moose density (average number of moose harvested/km^2^) (a-b), and number of scavenged carcasses between kills and distance (km) from the kill site to the nearest road (c).

|  |  |  |  |  |
| --- | --- | --- | --- | --- |
| Parameter | β | SE | *95% CI* | |
|  |  |  |  |  |
| (a) Scandinavia – Spring |  |  |  |  |
|  |  |  |  |  |
| Intercept | 0.74 | 0.62 | -0.67 | 2.21 |
| Bear Presence (A:S) | -0.74 | 0.90 | -2.45 | 0.83 |
| Julian Date | -0.01 | 0.01 | -0.03 | 0.004 |
| Bear Presence x Julian Date | 0.02 | 0.01 | -0.003 | 0.04 |
| Pack Size | -0.21 | 0.08 | -0.39 | -0.03 |
| Moose Density | -0.18 | 0.10 | -0.42 | -0.03 |
|  |  |  |  |  |
| (b) Scandinavia – Summer |  |  |  |  |
|  |  |  |  |  |
| Intercept | -0.19 | 0.12 | -0.42 | 0.04 |
| Bear Presence (A:S) | 0.39 | 0.17 | 0.05 | 0.73 |
| Julian Date | -0.05 | 0.09 | -0.23 | 0.14 |
| Prey Type (N:NN) | 0.71 | 0.23 | 0.26 | 1.17 |
| Pack Size | 0.07 | 0.08 | -0.08 | 0.22 |
| Moose Density | -0.09 | 0.08 | -0.24 | 0.07 |
|  |  |  |  |  |
| (c) Yellowstone – Summer |  |  |  |  |
|  |  |  |  |  |
| Intercept | 1.40 | 0.06 | 1.29 | 1.51 |
| Bear Presence (A:P) | 0.11 | 0.05 | 0.01 | 0.20 |
| Julian Date | 0.07 | 0.02 | 0.03 | 0.12 |
| Prey Type (S:L) | 0.18 | 0.05 | 0.08 | 0.27 |
| Pack Size | -0.01 | 0.04 | -0.09 | 0.06 |
| Scavenge | 0.20 | 0.02 | 0.16 | 0.24 |
| Road | -0.01 | 0.02 | -0.06 | 0.03 |
|  |  |  |  |  |
|  |  |  |  |  |
